# Effects of Garlic *Allium sativum* Powder on Nutrient Digestibility, Haematology, and Immune and Stress Responses in Eurasian Perch *Perca* *fluviatilis* Juveniles

**DOI:** 10.3390/ani11092735

**Published:** 2021-09-19

**Authors:** Mahyar Zare, Hung Quang Tran, Marketa Prokešová, Vlastimil Stejskal

**Affiliations:** Faculty of Fisheries and Protection of Waters, South Bohemian Research Center of Aquaculture and Biodiversity of Hydrocenoses, Institute of Aquaculture and Protection of Waters, University of South Bohemia in České Budějovice, Husova třída 458/102, 370 05 České Budějovice, Czech Republic; mzare@frov.jcu.cz (M.Z.); htranquang@frov.jcu.cz (H.Q.T.); mprokesova@frov.jcu.cz (M.P.)

**Keywords:** aquaculture, cortisol, fish, haematology, immunology, myeloid cells, stress

## Abstract

**Simple Summary:**

Herbal medicine feed supplements are used as growth promoters, immune system stimulants, and to combat stress. We evaluated the effects of garlic powder in the diet of European perch. The inclusion of garlic powder was shown to improve whole body composition, feed digestibility, and biochemical and immunohematological effects, and increased resistance against overcrowding stress.

**Abstract:**

The supplementation of fish diets with phytogenics can increase growth performance and can modulate immune system response. European perch *Perca fluviatilis* (initial weight 25.0 ± 0.4 g) were fed a diet including 0 (Control), 10 (G10), 20 (G20), and 30 (G30) g kg^−1^ garlic powder. No significant difference in the growth parameters and somatic indices were observed. Significantly higher fat digestibility was observed in G10 and G30 diets compared to in the control and G20 diets(*p* < 0.05). Significantly greater red blood cell and white blood cell counts were observed with the G10 diet (*p* < 0.05). Garlic significantly decreased serum cholesterol in all of the experimental groups. Serum albumin was significantly higher in the G10 and G20 diets (*p* < 0.05). Immediately after the overcrowding stress challenge, the garlic groups showed significantly higher cortisol levels than the control group, while no significant difference was observed in the glucose concentration among groups. At 1 h post-stress, all of the groups that had been fed a garlic-supplemented diet showed lower cortisol levels than the control group, and this trend was maintained at 6 and 24 h post stress (*p* < 0.05), and glucose level in all garlic groups was significantly lower than control (*p* < 0.05). Garlic at 10 g kg^−1^ in feed can improve apparent fat digestibility and selected blood parameters and can enhance resistance against high-density and net handling stress in Eurasian perch.

## 1. Introduction

Commercial production of fish, shellfish, and seafood is projected to increase by approximately 62% by 2030 [1]. Thus, the aquaculture rearing system is changing from an extensive system to intensive and semi-intensive systems [2], which might increase the chance of infectious disease outbreaks occurring [3]. Botanical derivatives and extracts, also known as phytogenics, have been used in fish diets as natural growth promoters and as immune stimulants. Currently, many plant extracts are considered as safe and cost-effective additives to aquafeed [4]. Antibiotics that can control pathogens on fish farms present concerns with respect to consumer health, animal welfare, and environmental pollution [5].

Garlic *Allium sativum* belongs to the *Liliaceae* family [6]. It has long been used as a herbal medicine and may be relevant to aquaculture because of its immunostimulant properties [7]. Garlic contains alliin, which has sulfur compounds including gamma-glutamyl-s-allyl-cysteine and S-allyl-L-cysteins sulfoxides. Moreover, garlic contains allicin (diallyl thiosulfate), which is responsible for garlic’s typical pungent smell and its medicinal properties [6], and other bioactive compounds, including vitamins (ascorbic acid, thiamine and riboflavin), minerals (potassium, phosphorus, calcium, magnesium, sodium, iron, selenium, and germanium), flavonoids (phenolic acids) [8], and amino acids [9]. Garlic powder has been reported to promote growth in Japanese seabass (*Lateolabrax japonicus*) [10] and rainbow trout *Oncorhynchus mykiss* [11] and to improve body composition in brown trout (*Salmo caspius*) [12]. However, garlic powder was not shown to improve growth performance in Asian sea bass (*Lates calarifer*) [13] or rohu (*Labeo rohita*) [14]. In addition, it has been reported to increase apparent nutrient digestion in rainbow trout [15] and Nile tilapia (*Oreochromis niloticus*) [16]. Dietary garlic powder has shown a favourable effect on blood total protein, albumin, and phagocytic activity in rainbow trout [17,18]; this plant increased fish resistance to ammonia stress [19]. Garlic powder increased immunoglobulins in European seabass (*Dicentrarchus labrax*) [20]. Garlic microencapsulated extract improved growth performance, body proximate composition, and biochemical and immunohematological parameters in rainbow trout (*Oncorhynchus mykiss*) [21]. These studies, regardless of the form of garlic presentation within diets, suggest that garlic may be used as an alternative to antibiotics [22].

Eurasian perch (*Perca fluviatilis*) is a carnivorous percid fish inhabiting northern Eurasia [23]. It is a domesticated species with a wide habitat range, and can be found in brackish water, estuaries, and rivers in recent decades [24], showing potential for European inland culture [23]. Eurasian perch can be a suitable species for recirculation aquaculture system (RAS) production and intensive culture [25], but handling through counting, sorting, and tank cleaning as well as high density stocking, may potentially increase energy consumption and decrease feed intake and growth [26]. 

Regarding to use of Eurasian perch in RAS culture in recent decades [25], the aim of the present study was to investigate the effects of garlic powder in feed on growth performance, body proximate composition, apparent nutrient digestibility, selected blood and immune parameters, and resistance to high-density and net handling stress in Eurasian perch juveniles.

## 2. Materials and Methods

### 2.1. Ethics Approval

The experimental procedures were performed under guidelines of the European Communities Directive (No. 2010/63/EU) on the protection of animals used for scientific purposes and have been approved by the Czech Ministry of Health (MSMT–6744/2018–2).

### 2.2. Preparation of Garlic Powder and Feed 

Garlic powder was purchased from EQUISERVIS, Prague, in Czech Republic. Garlic powder was produced by Pommier Nutrition, Thymerais—France. (Accessed: 4 June 2019) (www.pommier-nutrition.com). Experimental feeds (Table 1) were extruded at Exot Hobby s.r.o. (Černá v Pošumaví, Czech Republic), packed in plastic vacuum bags, and stored at 4 °C until use. In the present study, corn meal was replaced by 10, 20, and 30 g garlic powder per kilogram of diet feed. The proximate composition of the basal diet, including dry matter, crude protein, crude fat, and ash, was 93.48%, 47.20%, 16.33%, and 8.91%, respectively (Table 1).

### 2.3. Experimental Design

Eurasian perch juveniles with an initial weight of 25.0 ± 0.4 g were obtained from Anapartners s.r.o fish farm (Prague, Czech Republic). Fish were transferred to the aquaria at the Institute of Aquaculture and Protection of Waters (České Budějovice, Czech Republic) and were fed a basal diet formulation (Table 1) without garlic powder for a 14-day acclimation period before the feeding trial [12]. After adaptation, 1320 fish were randomly distributed into twelve 150 L tanks (110 fish per tank) with a water flow rate 10 L min^−1^ in RAS [25]. Each diet was tested with three replicates. Fish were fed manually to apparent satiation at 08:00, 12:00, and 16:00 for 87 days, and uneaten feed was collected at maximum of 30 min after each meal. Water temperature, pH, and dissolved oxygen (DO) were measured daily by an HQ40D portable multi-meter (Hach Lange GmbH, Düsseldorf, Germany) and were maintained at 22.1 ± 0.5 °C, pH 7.14 ± 1.61, and DO 8.16 ± 0.42 mg L^−1^, respectively. The photoperiod was 12L:12D [27].

### 2.4. Growth Performance

At the end of the feeding trial, feed was withheld for 24 h. The fish were anesthetized by tricaine methane sulphonate (MS-222) at 200 mg L^−1^ water [21] and counted, and individual length and weight were measured. Growth performance and survival rate were calculated [21]. Final body weight (FBW), feed intake (FI), weight gain (WG), weight gain percentage (WG%), feed conversion ratio (FCR), specific growth rate (SGR), protein efficiency ratio (PER), survival rate (SR%), condition factor (CF%), hepatosomatic index (HSI), and viscerosomatic index (VSI) were calculated as follows:

FBW (g) = Final body weight
WG (g) = [Final body weight (g) − Initial body weight (g)]
WG% = [Final body weight (g) − Initial body weight (g)]/initial body weight (g) × 100
FI (g/fish) = dry feed consumed/number of fish
PER = Weight gain (g)/total protein intake (g)
FCR = dry feed intake (g)/WG (g)
SGR (% day^−1^) = 100 × [ln final body weight (g) − ln initial body weight (g)]/time (days)
SR% = (number of fish after test/initial number of stocked fish) × 100
CF% = [fish body weight (g)/(fish length)^3^(cm)] × 100
HSI% = [liver weight (g)/body weight (g)] × 100
VSI% = [viscera weight (g)/body weight (g)] × 100

### 2.5. Whole Body Proximate Composition

At the end of experiment, two fish per each tank (*n* = 6 per group) were randomly selected. Fish were anesthetized with MS-222 at 200 mg L^−1^ water [21] and were killed by a sharp blow to the head. The entire fish body was ground, packed individually into plastic bags, and stored at −20 °C for whole body proximate composition analysis. Both body and feed proximate composition analyses were conducted according to the methods of the Association of Official Analytical Chemists (AOAC) [28]. Crude fat was analyzed by the extraction method using hexane–isopropanol (3:2) according to Hara and Radin, [29] with slight modifications [30]. Crude protein was measured using the Kjeldahl method (BUCHI Labortechnik AG, type K-360, Königswinter, Germany) [12]. Dry matter was analyzed by drying in a NÜVE type FN 400P oven (NÜVE, Ankara, Turkey) at 105 °C to a stable weight [21]. Ash was analyzed in a muffle furnace L 40/11 BO (Nabertherm GmbH, Lilienthal, Germany)) at 550 °C for 4 h [12].

### 2.6. Apparent Digestibility Coefficients

Diets contained 5 g kg^−1^ yttrium oxide (Y_2_O_3_) as an inert marker (Table 1). Two hours after the final daily meal, the tanks were brushed and cleaned, and the remaining faeces were discarded. After cleaning, the faeces were collected overnight by sieving every two hours from outlets until the first daily feeding in the morning. The faeces from each tank were stored separately at −20 °C after centrifugation at 3000 rpm for 15 min for analysis [31]. The Y_2_O_3_ in the diet and faeces was measured using inductively coupled plasma emission spectrometry following digestion with nitric acid at 180 °C for 48 h. The apparent digestibility coefficients (ADC) of the dry matter, protein, and fat were calculated with the following formula [15]:

% digestibility = 100 × 100 [(yttrium in feed/yttrium in faeces) × (nutrient in faeces/nutrient in feed)].

### 2.7. Haematology and Biochemistry

After 24 h starvation to induce the post-absorptive condition, two fish from each tank (*n* = 6 per group) were randomly netted and anesthetized by tricaine methane sulphonate (MS-222) at 200 mg L^−1^ water [21]. Duplicate blood samples were drawn from the caudal vein into heparinized and non-heparinized sterile syringes. The heparinized blood samples were transferred to heparinized Eppendorf tubes and were placed on ice for haematological analysis. The non-heparinized blood samples were transferred to non-heparinized Eppendorf tubes, and samples were left on ice for 2 h to clot [32]. The serum was subsequently separated by centrifugation (Heraeus Megafuge 16 R Centrifuge) at 4200 rpm for 15 min at 4 °C and was stored at −80 °C until analysis [33]. Red blood cells (RBC), white blood cells (WBC), and subpopulations were counted according to a modified method of Korytář et al. [34]. The heparinized blood (10 µL) was diluted in 200 µL RPMI medium on ice for cell composition evaluation by a FACS Canto flow cytometer (Heidelberg, Germany). The biochemical parameters of the blood were assessed using an Abbott Architect c8000 clinical chemistry analyzer (Abbott, Chicago, IL, USA) and assay kits [19] according to manufacturer’s instructions, as follows: serum total cholesterol, B7D6C7 G3-5321/R02 (Abbott, Chicago, IL, USA); triglycerides B7D7E7 G3-9334/R03 (Abbott, USA); alanine aminotransferase (ALT), B8L9x7 G5-4432/R05 (Abbott, USA); aspartate aminotransferase, G8-1502/R06 B8LY7 (Abbott, USA); albumin, 7D53-2030-3927/R6 (Abbott, USA); total protein, G6-6667/R04 B7D7D7 (Abbott, USA); and glucose, B3L8X7 G3-5375/R02) (Abbott, USA). Cortisol levels were analyzed with a cortisol assay kit (L2KCO2) using an immunochemistry analyzer Immulite 2000Xpi Siemens (Siemens Healthcare GmbH, Erlangen, Germany) at the Stafila laboratory, České Budějovice, Czech Republic.

### 2.8. Respiratory Burst and Phagocytic Activity

Two fish per tank (*n* = 6 per group) were anesthetized with MS-222 (200 mg L^−1^ water) [21]. The head kidney was removed, and the leukocytes were separated by pushing them through a nylon sieve with RPMI-1640 medium, according to the method by Biswas et al. [35]. A respiratory burst activity assay was conducted using nitro blue tetrazolium with minor modifications according to Zaineldin et al. [36]. Briefly, the leukocyte suspension was transferred into 96-well plates, and an equivalent volume of 0.2% nitro blue tetrazolium solution (Sigma, Ronkonkoma, NY, USA) was added to each well and was incubated for 30 min at room temperature. After incubation, N-dimethylformamide (Sigma, USA) was added and centrifuged for 5 min at 3000 rpm. The respiratory burst activity was reported as the mean fluorescence intensity. 

Phagocytic activity was assessed using a modified method from Morimoto et al. [37]. The leukocytes of head kidney were separated by washing with PBS (5 × 10^5^ cells mL^−1^) and were incubated with latex beads at 25 °C for 2 h, after which cell-related fluorescence was evaluated, and the samples were transferred into 96-well plates and assessed with a FACS Canto flow cytometer (Heidelberg, Germany) to detect the fluorescence of the beads engulfed by the phagocytic cells.

### 2.9. High-Density and Net Handling Stress Challenge

At the end of the feeding period, working with one tank at a time, the volume of water was decreased to leave the fish in a high-density condition (0.67 kg/L) for one minute with adequate aeration to avoid additional stress [38]. The fish were netted and removed from the water for 30 s [27] and were then returned to the tank, where the water level of the tank was increased back to the original volume, and the density was reduced [38]. Immediately after, two fish per tank were randomly selected (*n* = 6 per group), and the tank was refilled. The fish were anaesthetized with MS-222, and 1 mL of blood was drawn from the caudal vein with non-heparinized sterile syringes. All of these fish were killed after sampling. Sampling was conducted prior to the stress challenge, immediately after stress, and 1, 6, and 24 h post-stress [27]. Blood sampling in each tank was completed within 5 min.

### 2.10. Statistical Analysis

All statistical analyses were performed using IBM SPSS Statistics v. 22 (Armonk, NY, USA). Data normality and homogeneity were checked using the Kolmogorov–Smirnov test. Data were analyzed by one-way ANOVA. Significant differences among the mean values was set at *p* < 0.05 using the Duncan test. In addition, to determine if the effect was linear and/or quadratic, a follow-up trend analysis using orthogonal polynomial contrasts was performed. The results are presented as mean ± SD (standard deviation of the mean).

## 3. Results

### 3.1. Growth Performance and Feed Utilization

No significant differences in growth performance and feed utilization, including final weight, weight gain, weight gain percent, specific growth rate, feed intake, feed conversion ratio, protein efficiency ratio, or survival rate, were observed among the groups (*p* > 0.05). The condition factor was significantly lower in the G30 diet group compared to the other groups (*p* < 0.05). In particular, the condition factor significantly linearly decreased with the increasing dietary garlic powder levels (*p* = 0.04). In addition, no significant differences in the viscerosomatic or hepatosomatic index were found (*p* > 0.05). (Table 2).

### 3.2. Body Proximate Composition

No significant differences were observed the among groups in terms of whole body dry matter, fat, or ash (*p* > 0.05). The level of protein in fish consuming the G30 diet was significantly higher than the G1 group (*p* < 0.05), but there were no significant differences among the controls, G10, and G20 groups (*p* > 0.05) or among the controls, G20, and G30 groups (*p* > 0.05). There was a significant linear (*p* = 0.01) and quadratic (*p* = 0.04) trend regarding the dietary garlic powder level for body protein content, where body protein content decreased with the inclusion of garlic powder at G10 and then increased with the inclusion of garlic powder at G20 and G30. (Table 3).

### 3.3. Apparent Digestibility Coefficient (ADC%)

Significantly higher dry matter digestibility was observed in all of the garlic-supplemented groups compared to the controls (*p* < 0.05). Moreover, significantly higher fat digestibility was found in the G10 and G30 groups compared to the control and G20 groups (*p* < 0.05). No differences in protein digestibility were observed among the groups (*p* > 0.05). A positive linear (*p* = 0.00) and quadratic (*p* = 0.00) trend was found for dietary garlic powder levels and protein digestibility, where protein digestibility increased with the inclusion of garlic powder at G10 and then decreased with the inclusion of garlic powder at G20 and G30. (Table 4).

### 3.4. Haematology and Serum Biochemistry

The number of RBCs and WBCs in G10 were significantly higher than those observed in the other groups (*p* < 0.05). The WBCs had positive quadratic trend (*p* = 0.01) to the dietary garlic powder and reached a peak in the G10 group. The RBCs had positive quadratic trend to the dietary G10 group (*p* = 0.01). (Table 5).

No significant differences in blood serum ALT and AST activity, triglycerides, or total protein were observed among the groups (*p* > 0.05). At all levels, garlic powder was associated with significantly lower levels of cholesterol (*p* < 0.05). Significantly higher levels of albumin were detected in the G10 and G20 groups compared to in the other groups (*p* < 0.05). A significant linear trend (*p* = 0.00) was observed regarding the dietary garlic powder level for albumin, where albumin increased with the inclusion of garlic powder at G10 and then decreased with the inclusion of garlic powder at G20 and G30. (Table 6).

### 3.5. Respiratory Burst and Phagocyte Activity 

Garlic powder inclusion did not affect respiratory burst activity (*p* > 0.05) or lymphocyte and myeloid cell phagocytic activity and index (*p* > 0.05) (Table 7). 

### 3.6. High-Density and Net Handling Stress Challenge

**Figure 1:**
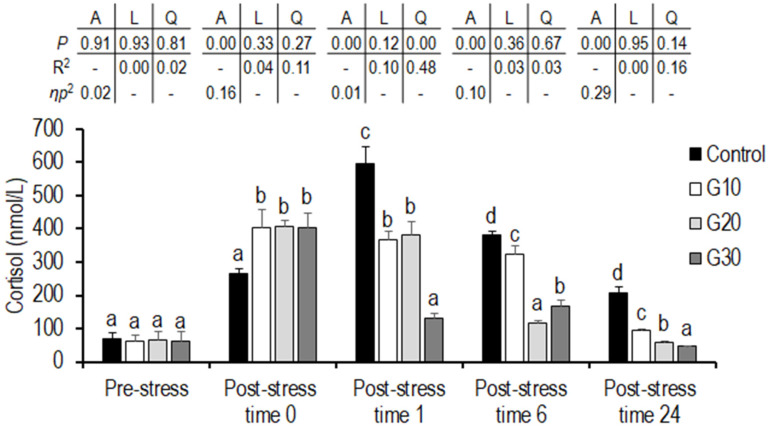
Serum cortisol of Eurasian perch provided feed supplemented with garlic powder under high-density and net handling stress. Values are presented as (mean ± SD; *n = 6*). Mean values with different superscripts within each time vary significantly according to one-way ANOVA (*p* < 0.05). A = the variance analysed by one-way ANOVA; L = the linear trend analysed by orthogonal polynomial contrasts; Q = the quadratic trend analysed by orthogonal polynomial contrasts. R^2^ = R square. *ƞ*_p_^2^ = partial eta squared. Control: without garlic supplement; G1: 10 g garlic powder per 1000 g diet; G2: 20 g garlic powder per 1000 g diet; G3: 30 g garlic powder per 1000 g diet. Pre-stress: before stress; Post-stress time 0: immediately after stress; Post-stress time 1: one hour after stress; Post-stress time 6: 6 h after stress; Post-stress time 24: 24 h after stress.

**Figure 2:**
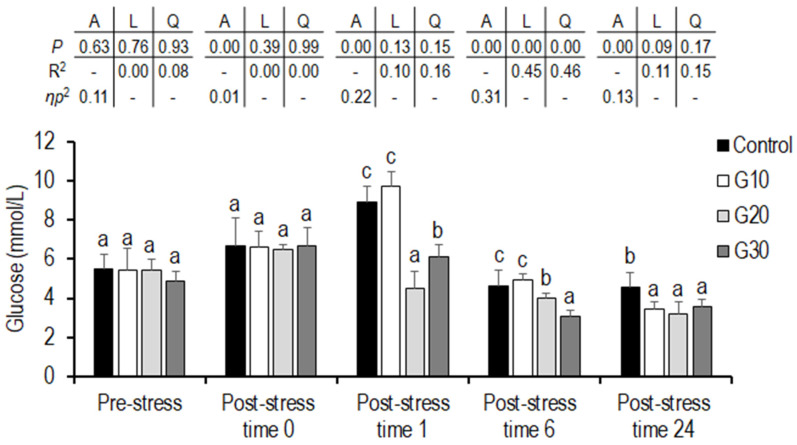
Serum glucose of Eurasian perch provided feed supplemented with garlic powder under high-density and net handling stress. Values are presented as (mean ± SD; *n* = 6). Mean values with different superscripts within each time vary significantly according to one-way ANOVA (*p* < 0.05). A = the variance analysed by one-way ANOVA; L = the linear trend analysed by orthogonal polynomial contrasts; Q = the quadratic trend analysed by orthogonal polynomial contrasts; R^2^ = R square. *ƞ*_p_^2^ = partial eta squared. Control: without garlic supplement; G1: 10 g garlic powder per 1000 g diet; G2: 20 g garlic powder per 1000 g diet; G3: 30 g garlic powder per 1000 g diet. Pre-stress: before stress; Post-stress time 0: immediately after stress; Post-stress time 1: one hour after stress; Post-stress time 6: 6 h after stress; Post-stress time 24: 24 h after stress.

No significant differences in the level of serum cortisol and glucose were observed among the groups before stress (*p* > 0.05). Immediately after stress, all garlic diet groups showed significantly higher levels of cortisol compared to the control group (*p* < 0.05). No significant differences in glucose levels were observed among the groups (*p* > 0.05). At 1 h, significantly higher cortisol was observed in the controls, and there was a significantly lower level in the G30 group compared to in the other groups (*p* < 0.05). At 1 h, a positive quadratic trend was found between the increasing levels of garlic powder and serum cortisol (*p* = 0.00), where serum cortisol decreased with the inclusion of garlic powder at G10, increased with the inclusion of garlic powder at G20, and then decreased with the inclusion of garlic powder at G30. At 1 h, the control and G10 groups showed significantly higher glucose compared to in the G20 and G30 groups, while the glucose level of the G20 group was significantly lower than that of the other groups (*p* < 0.05). At 6 h, significantly higher and lower levels of cortisol were observed in the control and G20 groups, respectively, compared to in the other groups (*p* < 0.05), where the highest level of glucose was found in the control and G10 groups, and the lowest level of glucose was found in the G30 group at significant levels (*p* < 0.05). At 6 h, a significant linear trend (*p* = 0.00) regarding the dietary garlic powder level was observed for serum glucose. With increasing levels of garlic powder, the serum glucose decreased linearly. A positive quadratic trend was observed between the increasing levels of garlic powder and the serum glucose, where the serum glucose increased with the inclusion of garlic powder at G10 and then decreased with the inclusion of garlic powder at G20 and G30. At 24 h, significantly higher and lower cortisol levels were detected in the control and G30 groups, respectively (*p* < 0.05), while glucose was significantly higher in the control group than in the garlic-fed groups (*p* < 0.05) (Figure 1 and Figure 2).

## 4. Discussion

In the present study, the inclusion of garlic powder in compound diets for European perch did not show significant effects on growth performance. This finding agrees with Sahu et al. [14], who reported that garlic powder in the diet of rohu at 1, 5, and 10 g kg^−1^ feed did not significantly improve SGR or FCR. Another report documented that the use of garlic powder at the level of 40 g kg^−1^ in European sea bass did not have a significant effect on final weight, while 60 g kg^−1^ significantly decreased final weight, specific growth rate, and feed intake [39]. In contrast, garlic powder improved growth performance in Japanese sea bass at 25 g kg^−1^ [10], in brown trout at 20 and 30 g kg^−1^ [12], and in European sea bass at 10 g kg^−1^ [40]. Enhanced growth performance can be attributed to garlic bioactive compounds, including alliin, allicin, and organosulfur compounds, especially thiosulfinates [8], which increase digestion, nutrient uptake, and growth [16]. Differences among the results can be related to differences in the experimental design, fish species [10,12,40], fish size [39,40], garlic supplement type (powder or extract), and its purity [41,42] and garlic supplement level in the diet [18,39].

The liver is active in fish metabolisms, and HSI can be a marker of the harmful effects from the environment or diet [43]. In our research, the HSI and VSI indices did not differ among groups. This agrees with Shalaby et al. [16], who reported no effect of garlic powder at 10, 20, 30, and 40 g kg^−1^ feed on HSI in Nile tilapia. In contrast, 30 g kg^1^ garlic powder in the diet of brown trout [12] and 32 g kg^−1^ in the diet of Nile tilapia [42] were associated with significantly decreased HSI. In contrast, the inclusion of 10 g kg^−1^ garlic powder in the diet of brown trout also significantly increased HSI and VSI [12]. Furthermore, Lee et al. [44] confirmed that 5 g kg^−1^ of garlic extract did not have an effect on HSI in sterlet (*Acipenser ruthenus*) after 5 weeks, but 5 g kg^−1^ of garlic extract increased the somatic index (HSI) in sterlet after 10 weeks. Moreover, the use of garlic powder at levels 5, 10, 15, 20, and 30 g kg^−1^ in the sterlet diet significantly decreased HSI after a 12-week feeding trial in all garlic groups [45]. These reports showed that feeding trial duration has a strong effect on the hepatosomotic index. In contrast, our results confirm that no significant difference in HSI and VSI among groups can be significantly related to non-accumulation fat in the whole body and liver [40,46] or reduced fat accumulation in the whole body and liver in the garlic groups [21,42].

The biological characteristics of fish along with environmental parameters, feeding protocols, and parasitic infections, affect the fish condition factor [47]. In recent studies, the addition of garlic powder to brown trout feed [12] did not increase the condition factor. In the present study, the condition factor in the G30 group was significantly reduced. Lower levels—10 g kg^−1^ garlic powder in Japanese sea bass [10] and 20 g kg^−1^ in sterlet [45] feed—significantly increased the condition factor, suggesting increased diet palatability [10,45]. In contrast, garlic powder at levels of 10, 20, and 30 g kg^−1^ significantly decreased the condition factor in Indian major carp, which is in line with our results [48]. In our study, the decrease in the condition factor can be attributed to the pungent odour of garlic in G30, which may have reduced feed palatability [49] and feed intake [39]. Moreover, previous reports proved that use of garlic powder in levels of 25 g kg^−1^ in the diet of Japanese seabass [10] and 60 g kg^−1^ [39] and 20 g kg^−1^ of garlic powder in European sea bass feed [40] decreased feed intake. In the present study, feed intake decreased in the G30 groups and subsequently decreased the condition factor [48] for Eurasian perch. 

The whole-body proximate composition of perch fed garlic powder did not show significant differences in dry matter, fat, or ash, while the G30 diet significantly increased body proximate protein. These results are comparable to those with 30 g kg^−1^ garlic powder in brown trout [12] and 30 g kg^−1^ in monosex redbelly tilapia (*Tilapia zilli*) [50], which improved body proximate protein composition. The inclusion of garlic powder in the diet of European seabass [40] and Nile tilapia [16,42] improved body proximate composition. Studies have shown that garlic supplementation can increase body proximate protein. Increasing protein and decreasing fat can be attributed to the organosulfur compounds found in garlic such as allicin, S-allyl cysteine, and diallyl-di-sulfide, which reduce fat aggregation in the body [42] due to the increasing bile acids in the garlic treatments [51]. Bile acids are considered to be regulatory molecules, and they have been considered to stimulate specific nuclear receptors in cells in the liver and gastrointestinal tract [52]. Increased protein can be interpreted as a result of the essential amino acids contained in garlic [9], increasing free amino acids in the muscle and resulting in protein synthesis [40]. 

Plant ingredients in fish diets can balance some micronutrients or bioactive compounds [53]. The evaluation of the digestibility coefficients of feed ingredients specify the nutrient utilization for different fish species [54]. At our lowest test level, garlic powder significantly improved dry matter and fat digestibility. Esmaeili et al. [15] observed higher dry matter, fat, and protein digestibility in rainbow trout fed with 30 g kg^−1^ of garlic powder in feed. Shalaby et al. [16] demonstrated that 30 g kg^−1^ of garlic powder increased protein and fat digestibility in Nile tilapia, similar to our results in perch. Other studies have confirmed that garlic powder improved the digestibility of nutrients and SGR and decreased FCR in European seabass at 20 and 30 g kg^−1^ [40], in Nile tilapia at 32 g kg^−1^ [42], and in rainbow trout at 0.5, 1, 5, and 10 g kg^−1^ [18]. Moreover, we found some studies showing that the use of 10 g kg^−1^ of garlic powder in the diet of sobaity sea bream (*Sparidentex hasta*) [55] and 5, 10, 15, and 20 g kg^−1^ of garlic powder in the diet of Asian sea bass significantly improved nutrient digestibility, SGR, and FCR [13]. Furthermore, the administration of microencapsulated garlic extract in rainbow trout at a level of 10 g kg^−1^ improved nutrient digestibility, SGR, and FCR as well [21]. These reports reveal that the administration of garlic as either a powder or an extract in different fish species increases growth performance [21,55] and nutrient digestibility due to the bioactive compounds found in garlic, such as allicin, which improved growth performance and nutrient digestibility in Nile tilapia [16,42] and European sea bass [40].

Red blood cell and withe blood cell counts are good key indices for evaluating fish physiology and pathology [56]. In our research, the administration of garlic at 10 g kg^−1^ increased RBC and WBC numbers compared to the other groups. Garlic powder has shown similar results in rainbow trout at 0.5, 1, 5, and 10 g kg^−1^ [18] and in rohu at 10 g kg^−1^ [14]. Nya and Austin [18] reported that 10 g kg^−1^ of garlic powder increased the WBCs in rainbow trout but did not affect RBC numbers. In contrast, the administration of 10 g kg^−1^ of garlic extract (allicin) in the diet of rainbow trout increased RBC numbers, but significantly decreased WBCs [41]. The use of garlic powder did not alter RBC and WBC numbers in brown trout at 10, 20, or 30 g kg^−1^ [12] or in beluga (*Huso huso*) at 10 g kg^−1^ [57], and it had no effect on RBC numbers in European sea bass at 10, 20, or 30 g kg^−1^, while 30 g kg^−1^ of garlic powder increased the WBCs in sea bass [40]. The higher number of WBCs found in perch in our study may be related to the immunostimulatory effect of garlic compounds on the kidney, spleen, and thymus [58], as reported in previous studies [13,18]. RBCs play important roles in oxygen transfer, decreasing hypoxia stress, and contributing to fish health [59]. Our findings of higher RBC counts can be attributed to the effect of garlic compounds such as allicin [41] on the head kidney as the main erythropoietic site in teleost fish [60]. In our study, diets containing garlic powder did not increase concentrations of blood lymphocytes or myeloid cells. This result is similar to the inclusion of 5, 10, 15, and 20 g kg^−1^ in the diet of Asian sea bass [13]. Nya et al. [41] reported that 10 g kg^−1^ of allicin in the diet of rainbow trout increased neutrophil concentration but showed no effect on lymphocyte and monocyte percentage. WBCs, including lymphocytes [61] and myeloid cells [62], have key functions against pathogens as a first line of defence [63]. Myeloid cells include neutrophils and eosinophils (granulocytes) along with monocytes (macrophages) in fish [62].

Fish health can be evaluated by blood serum biochemical parameters [33], specifically the levels of ALT and AST [21,55], which are affected by diet, environment, and stress [64]. The level of ALT and AST activity is considered an indicator of liver health [33]. The levels of blood serum ALT and AST can be affected by stocking density [65]; water parameters [66]; and fish species [55,57], age, and sex [67]. In the present study, garlic powder did not show significant effects on serum ALT and AST activity. In agreement with our results, garlic powder in the 40 g kg^−1^ diet did not show significant effect on ALT and AST activity in Asian sea bass (*Lates calcarifer*) [68]. Furthermore, a mixture of cumin seeds (*Nigella sativa*) and turmeric (*Curcuma longa* Linn.) powder at the levels of 5 and 10 g kg^−1^ feed (1:1 w/w) did not show significant difference in the levels of ALT and AST in the Asian sea bass (*L. Calcarifer*), which is the same as in our study [69]. Other studies showed no effect on ALT activity in sobaity sea bream [55] or beluga at 10 g kg^−1^ feed [57]. Serum AST activity significantly increased in sobaity sea bream with 10 g kg^−1^ of garlic [55] and decreased in beluga [57]. Garlic powder at 32 g kg^−1^ [42] and 30 and 40 g kg^−1^ significantly decreased blood serum ALT and AST activity in Nile tilapia [16]. Moreover, garlic powder at the levels 5, 10, and 15 g kg^−1^ in feed decreased the level of blood serum ALT and AST significantly in common carp (*Cyprinus carpio*) [70]. In contrast, the inclusion of 40 and 50 g kg^−1^ of garlic powder significantly increased blood serum ALT and AST activity in rainbow trout [33]. The present study showed that the levels of ALT and AST can at least be related to fish species and to herbal medicine level and species [68,70] in the diet, similar to previous studies [55,69]. Moreover, no significant difference in the level of blood serum ALT and AST in our experimental fish, indicating that 10, 20, and 30 g kg^−1^ of garlic powder in perch diet were safe doses, as they did not disturb liver finction, as confirmed in the previous studies [68,69]. The reduction of ALT and AST activity in the blood serum can be attributed to the antioxidant compounds found in garlic, including S-allyl cysteine and diallyl-di-sulfide [71] and the flavonoids rutin, tangeretin, and nobiletin [72]. These antioxidant compounds hinder fat peroxidation in the cell membrane and prevent ALT and AST secretion into the blood [55].

Triglyceride and cholesterol were measured as blood serum biochemical parameters [55]. We observed no significant differences in the triglyceride levels among groups, while cholesterol was significantly lower in the garlic-fed groups. Garlic powder at 5, 10, 15, and 20 g kg^−1^ feed reduced cholesterol and triglycerides in Asian sea bass [13] as well as in rainbow trout at 20, 30, and 50 g kg^−1^ [33]. In contrast, 10 g kg^−1^ garlic powder in feed increased cholesterol and triglyceride levels in sobaity sea bream [5]. Apparently, garlic sulphur compounds reduce triglyceride levels in the blood serum [42]. Allicin is a main bioactive compound found in garlic that is responsible for hypolipidemia and hypocholesterolemia [73] and inhibits cholesterol biosynthesis [74]. In this line, our result showed that garlic powder at the higher level of G30 (30 g garlic powder per kg feed) significantly decreased blood serum cholesterol levels in our experimental species. In line with our study, Shalaby et al. [16] confirmed that garlic powder improved nutrient digestibility, SGR%, and FCR and increased fat digestibilty. Moreover, garlic powder decreased whole body fat and blood plasma lipids in Nile tilapia (*O. niloticus*). In another research study that was of a similar design to ours, garlic powder improved SGR, FCR, and nutrient digestibility and decreased total blood serum lipids, triglycerides, and cholesterol in Asian sea bass [13]. Moreover, Adineh et al. [21] reported the use of microencapsulated garlic extract at the level of 10 g kg^−1^ feed in rainbow trout improved SGR%, FCR, and nutrient digestibility and decreased whole body fat, which is the same as our results. Another study showed that garlic oil (0.15 g kg^−1^ feed) and powder (32 g kg^−1^ feed) increased nutrient digestibility by improving SGR% and FCR and decreased fat accumulation in the whole body and in the blood serum triglycerides and cholesterol [42] like our study. Previous studies [13,16,21,42] confirm our results and have demonstrated that whole body fat accumulation, apparent fat digestibility, and levels of blood serum triglycerides and cholesterol are related. In fact, those studies confirmed that increasing fat digestibility decreases fat accumulation in the whole body and reduces blood serum triglycerides and cholesterol [16,42]. 

In the present study, blood serum albumin was significantly higher in the G10 and G20 groups. Albumin has a protein structure. Albumin is primarily produced in the liver and prevents blood from leaking out of blood vessels. Albumin also transfers medicines and other substances across the blood for tissue growth and is used for tissue growth and healing [75]. Garlic powder increased blood serum albumin in amur carp [76] and rainbow trout [18]. The inclusion of garlic powder at levels of 10, 20, and 30 g kg^−1^ in brown trout feed did not significantly increase blood serum albumin [12], but an increase was seen in Asian sea bass at the levels of 5, 15, and 20 g kg^−1^ feed [13]. These differences in results can be related to the garlic dose and fish species as well as feed ingredient composition.

Blood serum protein parameters specifically show the status of fish as they react to internal and external factors [42]. Blood serum protein provides energy, creates new cells, reconstructs muscles, transports other nutrients such as messengers in the body, and supports the immune system [70]. We did not find blood serum total protein to differ among groups. This was also reported by Talpur and Ikhwanuddin [13], who administered garlic powder to Asian sea bass at the levels of 5, 10, 15, 20 g kg^−1^ feed, and by Nya and Austin [17], who used 5 and 10 g kg^−1^ in the feed of rainbow trout. In contrast, garlic powder at 10 g kg^−1^ in the diet of sobaity sea bream [55] and at 20 g kg^−1^ in brown trout [12] increased blood serum total protein. Total protein indicates immune system status [77]. Increased blood serum protein in the garlic groups can be interpreted as a higher amount of amino acids in the garlic groups as well as higher amounts of sulfur compounds including S-allyl cysteine sulfoxide [9] and and stimulate liver to synthesize blood serum proteins [42].

Phytogenics enhance the immune system of fish [78], but in our study, garlic in the diet of perch did not improve respiratory burst activity. This finding is in agreement with Mahfouz et al. [79], who reported that 20 g kg^−1^ of garlic powder in Nile tilapia feed did not increase respiratory burst activity, which may be related to fish species, culture, and feeding conditions. Respiratory burst is a latent metabolic route in the cells and is activated upon pathogen exposure. It destroys pathogens through the synthesis of powerful oxidizing compounds [80]. The use of 5 and 10 g kg^−1^ of garlic powder in rainbow trout increased respiratory burst reactive oxygen species [17] and 15 g kg^−1^ in Amur carp (*Cyprinus carpio haematopterus*) diets [76] was shown to increase respiratory burst activity. Increasing superoxide anion production elevates reactive oxygen species [14]. The administration of 10 g kg^−1^ garlic powder to Asian sea bass [13] and 0.5 and 1 g kg^−1^ to rainbow trout [18] increased superoxide anion production (*p* < 0.05).

Phagocytic activity is considered to be an indicator of fish immune system activity [81]. We did not find the inclusion of garlic powder in the diet of Eurasian perch to be associated with the phagocytic activity of lymphocytes or myeloid cells, unlike another reports that indicate that garlic powder increased phagocytic activity and the phagocytic index in Nile tilapia at 10 and 20 g kg^−1^ [82], Asian sea bass at 20 g kg^−1^ [13], and rainbow trout at 10 g kg^−1^ [18]. Garlic extract (allicin) increased phagocytic activity in rainbow trout at 5 and 10 g kg^−1^ feed [41]. Fish species and the level of garlic can determine its effect on the immune system. The phagocytic boost of garlic powder or garlic extract [18,41] can be attributed to the immunostimulatory effect of compounds such as allicin [41], germanium, and lectin [83]. However, the present study showed that garlic powder cannot boost phagocytic activity, at least in perch. Although we did not find a significant immune response in our experimental fish in our study, immune response may happen during a longer feeding trial, at higher levels of garlic powder [14], or with the use of garlic extract in the diet [41]. In light of this, Sahu et al. [14] mentioned that superoxide anion production, which elevates reactive oxygen species was significantly higher in garlic groups compared to in control groups after 20-, 40-, 60- and 70-day feeding trials. However, the level of superoxide anion production after 60 days was higher than it was at 20, 40, and 70 days. This result shows that immune response can at the very least be related to feeding trial duration.

A mixture of 200 ppm garlic and labiatae essential oils (Delacon, Austria) (PHYTO diet) did not reduce blood plasma cortisol or glucose in European sea bass [84]. Garlic powder at 10, 20, and 30 g kg^−1^ feed in brown trout [12] and at 1, 5, and 10 g kg^−1^ in rohu [14] showed no significant effect on serum glucose, while it decreased levels of blood serum glucose at 5, 10, 15, and 20 g kg^−1^ in the feed of Asian sea bass [13] and 40 g kg^−1^ in Nile tilapia feed [16]. Zaefarian et al. [12] suggested that the efficacy of garlic supplementation intake can be related to culture conditions and fish species. The reduction of glucose in blood serum can be attributed to the effect of garlic organosulfur compounds such as alliin (S-allyl cysteine sulfoxide) [85] and diallyl trisulfide [86], which have been shown to stimulate insulin secretion in diabetic mice [85] and rats [86], respectively. Although increasing levels of amino acids elevate insulin secretion, especially in carnivorous fish [87], increasing blood glucose levels in fish also elevate insulin levels [88]. Garlic organosulfur compounds increase glycemic control through enhanced insulin secretion and increase insulin sensitivity [85].

Blood cortisol and glucose are considered primary and secondary stress indicators in fish [89]. Cortisol is the key circulating glucocorticoid in fish, and its level is indicated by its cytosolic receptor, which regulates the expression of genes involved in growth, metabolism, and immune function [90]. Cortisol, a common stress indicator increased blood glucose in response to stress [91]. 

In the present study, post-challenge, the observed blood serum cortisol was significantly higher in all of the garlic groups compared to the control group, while there was no difference in the serum levels (*p* > 0.05) among groups. Elevated blood serum glucose indicates a higher stress level, requiring fish to increase energy expenditure [92]. Along with serum cortisol, glucose increases in response to energy demands [93]. Under stress, catecholamines and cortisol exert an effect on hepatocytes and induce glycolysis and gluconeogenesis, leading to an increase serum glucose [94].

At 24 h post-stress, the G30 group showed lower blood serum cortisol and glucose compared to the other groups (*p* > 0.05). At 1, 6, and 24 h post-stress, blood serum cortisol was lower in all of the garlic groups compared to the levels in the control grpi. High-density stocking [95], handling [27], heat stress [96], and low water pH [66] have been reported to increase levels of cortisol and glucose in fish. The inclusion of 2 mg nano selenium and 2 ppm garlic extract reduced blood plasma cortisol and glucose in grass carp (*Ctenopharyngodon idella*) under stocking density stress [97], while 200 ppm of a mixture of garlic and labiatae essential oil (Delacon, Austria) (PHYTO diet) reduced blood serum cortisol after 2 h overcrowding stress but did not show any effect on blood glucose (*p* > 0.05) in European sea bass [84]. In the present study, lower cortisol and glucose may be attributed to the bioactive compounds found in garlic, including alliin and diallyl trisulfide [98], which were higher in the G30 diet compared to in the other diets [13,21,42].

## 5. Conclusions

Garlic powder at 10 g kg^−1^ diet shows beneficial effects on haematology, blood biochemical parameters, and the apparent digestibility of nutrients including fat. The inclusion of garlic at 30 g kg^−1^ improved whole-body protein composition and increased resistance against high-density and net handling stress in European perch.

Further research should include garlic *A. sativum* powder in the diets of European perch of different sizes and developmental stages to evaluate growth performance and haematological and immunological parameters, including digestive enzymes and liver antioxidant activity. We suggest further study to identify bioactive compounds in garlic that are effective in immune-related gene expression.

## Figures and Tables

**Table 1:** Proximate composition of experimental diets with and without garlic powder.

Ingredients (g kg^−1^)	Control	G10	G20	G30
Fish meal	271	271	271	271
Soybean concentrate	290	290	290	290
Corn meal	97	87	77	67
Soybean meal	128.5	128.5	128.5	128.5
Garlic powder ^a^	0	10	20	30
Fish oil	77	77	77	77
Rapeseed oil	58	58	58	58
Methionine ^b^	8	8	8	8
Lysine ^c^	5	5	5	5
Valine ^d^	2	2	2	2
L-Threonine ^e^	0.5	0.5	0.5	0.5
Vitamins & minerals ^f^	8	8	8	8
Binder ^g^	50	50	50	50
Yttrium oxide (Y_2_O_3_) ^h^	5	5	5	5
Proximate composition analysis				
Dry matter%	93.48	93.54	93.71	93.17
Crude protein%	47.20	46.84	46.59	46.33
Fat%	16.33	16.51	15.98	16.13
Ash%	8.91	8.78	8.78	8.82
Fiber%	1.82	3.87	3.98	2.42
Nitrogen-free extract (NFE) ^f,i^	19.22	17.54	18.38	19.47
Gross energy (Kj g^−1^) ^g,j^	17.90	18.24	17.99	17.72

^a^ Garlic powder was purchased from EQUISERVIS, Prague, in Czech Republic. ^b^ Adisseo, Shanghai, China; ^c^ Inner Mongolia Eppen Biotech Co., Ltd., Ningxia, China.; ^d^ Ajinomoto Animal Nutrition Europe; ^e^ Ningxia Eppen Biotech, China; ^f^ Aminovitan Sak, Trouw Nutrition Biofaktory s.r.o, Prague, Czech Republic; ^g^ binder (NutriBind, Adisseo, Shanghai, China) (3.0%); ^h^ yttrium oxide (Y_2_O_3_), Sigma, Ronkonkoma, NY, USA; ^i^ nitrogen-free extracts (NFE) = dry matter—(crude protein + crude lipid + ash + fiber) [15]; ^j^ gross energy was calculated according to following formula: gross energy (MJ/kg) = (protein × 23.6 kJ g^−1^) + (fat × 39.5 kJ g^−1^) + (carbohydrates × 17.2 kJ g^−1^) [15]; control: without garlic supplement; G10: 10 g garlic powder per 1000 g diet; G20: 20 g garlic powder per 1000 g diet; G30: 30 g garlic powder per 1000 g diet.

**Table 2:** Growth performance and feed utilization of juvenile Eurasian perch consuming feed supplemented with garlic powder.

							Linear Trend		Quadratic Trend	
Parameters	Control	G10	G20	G30	ANOVA	*ƞ*_p_^2^	*P*-Value	R^2^	*P*-Value	R^2^
Initial weight (g)	24.75 ± 0.47	25.37 ± 0.41	24.77 ± 0.13	25.18 ± 0.18	0.13					
Final weight (g)	66.29 ± 1.99	67.39 ± 1.89	64.89 ± 1.53	65.52 ± 2.93	0.55					
Feed intake (g fish^−1^)	68.49 ± 1.82	68.41 ± 1.06	68.00 ± 0.80	67.83 ± 1.76	0.92					
Weight gain (g)	41.53 ± 2.02	42.02 ± 1.51	40.12 ± 1.40	40.34 ± 3.09	0.65					
Weight gain%	167.82 ± 9.25	165.55 ± 3.67	161.97 ± 4.83	160.29 ± 13.38	0.71					
Feed conversion ratio	1.68 ± 0.15	1.64 ± 0.07	1.73 ± 0.08	1.71 ± 0.16	0.85					
Specific growth rate (% day^−1^)	1.12 ± 0.04	1.11 ± 0.01	1.10 ± 0.02	1.09 ± 0.05	0.76					
Protein efficiency ratio	1.25 ± 0.09	1.29 ± 0.03	1.24 ± 0.07	1.26 ± 0.13	0.90					
Survival rate (%)	96.36 ± 2.40	98.18 ± 0.91	96.96 ± 1.89	97.57 ± 2.78	0.75					
Condition factor%	1.25 ± 0.05 ^b^	1.24 ± 0.02 ^b^	1.18 ± 0.03 ^b^	1.10 ± 0.03 ^a^	0.00	0.78	0.04	0.33	0.05	
Hepatosomatic index%	1.59 ± 0.36	1.56 ± 0.28	1.53 ± 0.37	1.38 ± 0.30	0.44					
Viscerosomatic index%	12.06 ± 2.28	13.13 ± 2.17	12.88 ± 1.56	13.19 ± 1.85	0.49					

Values are presented as (mean ± SD; *n* = 110). Mean values with different superscripts within a row vary significantly according to one-way ANOVA (*p* < 0.05). R^2^ = R squared. *ƞ*_p_^2^ = partial eta squared. Control: without garlic supplement; G10: 10 g garlic powder per 1000 g diet; G20: 20 g garlic powder per 1000 g diet; G30: 30 g garlic powder per 1000 g diet.

**Table 3:** Body proximate composition of Eurasian perch consuming feed supplemented with garlic powder.

							Linear Trend		Quadratic Trend	
Parameters	Control	G10	G20	G30	ANOVA	*ƞ*_p_^2^	*P*-Value	R^2^	*P*-Value	R^2^
Dry matter%	32.08 ± 1.15	31.75 ± 1.33	32.10 ± 1.71	32.26 ± 1.09	0.92					
Fat%	11.16 ± 1.50	10.96 ± 1.44	10.78 ± 1.13	9.76 ± 1.24	0.29					
Protein%	17.42 ± 0.60 ^ab^	16.98 ± 0.69 ^a^	17.39 ± 0.98 ^ab^	18.31 ± 0.67 ^b^	0.04	0.33	0.01	0.23	0.04	0.25
Ash%	3.38 ± 0.43	3.01 ± 0.60	3.16 ± 0.19	3.53 ± 0.29	0.16					

Values are presented as (mean ± SD; *n* = 6). Mean values with different superscripts within a row vary significantly according to one-way ANOVA (*p* < 0.05). R^2^ = R square. *ƞ*_p_^2^ = partial eta squared. Control: without garlic supplement; G10: 10 g garlic powder per 1000 g diet; G20: 20 g garlic powder per 1000 g diet; G30: 30 g garlic powder per 1000 g diet.

**Table 4:** Apparent digestibility coefficient for dry matter, fat, and protein in Eurasian perch provided feed supplemented with garlic powder.

							Linear Trend		Quadratic Trend	
Parameters	Control	G10	G20	G30	ANOVA	*ƞ*_p_^2^	*P*-Value	R^2^	*P*-Value	R^2^
ADCd%	77.53 ± 0.59 ^a^	80.78 ± 0.50 ^b^	79.60 ± 0.87 ^b^	81.12 ± 1.83 ^b^	0.01	0.71	0.69	-	0.27	-
ADCf%	78.29 ± 0.46 ^a^	79.89 ± 0.68 ^b^	78.35 ± 0.72 ^a^	80.16 ± 1.08 ^b^	0.03	0.65	0.94	-	0.09	-
ADCp%	92.41 ± 0.30	93.33 ± 0.28	92.66 ± 0.32	92.49 ± 0.55	0.06					

Values are presented as (mean ± SD; *n* = 6). Mean values with different superscripts within a row vary significantly according to one-way ANOVA (*p* < 0.05). R^2^ = R squared. *ƞ*_p_^2^ = partial eta squared. Control: without garlic supplement; G10: 10 g garlic powder per 1000 g diet; G20: 20 g garlic powder per 1000 g diet; G30: 30 g garlic powder per 1000 g diet; ADCd: Apparent digestibility coefficient of dry matter. ADCf: Apparent digestibility coefficient of fat. ADCp: Apparent digestibility coefficient of protein.

**Table 5:** Haematological parameters of Eurasian perch fed with feeds supplemented with garlic powder.

							Linear Trend		Quadratic Trend	
Parameters	Control	G10	G20	G30	ANOVA	*ƞ*_p_^2^	*P*-Value	R^2^	*P*-Value	R^2^
Red blood cells (*n* × 10^6^ μL^−1^)	283,896 ± 77,236 ^ab^	464,543 ± 78,157 ^c^	256,285 ± 16,266 ^a^	352,395 ± 46,442 ^b^	0.00	0.68	0.13	-	0.01	0.34
White blood cells (*n* × 10^6^ μL^−1^)	19,711 ± 5397	30,589 ± 7884 ^b^	18,520 ± 4312 ^a^	21,245 ± 5152 ^a^	0.00	0.44	0.03	0.19	0.01	0.33
Lymphocytes (%)	91.84 ± 3.51	89.77 ± 4.56	93.91 ± 1.84	94.11 ± 2.87	0.11					
Myeloid cells (%)	8.15 ± 3.51	10.22 ± 4.56	6.08 ± 1.84	5.88 ± 2.87	0.11					

Values are presented as (mean ± SD; *n* = 6). Mean values with different superscripts within a row vary significantly according to one-way ANOVA (*p* < 0.05). R^2^ = R square. *ƞ*_p_^2^ = partial eta squared. Control: without garlic supplement; G10: 10 g garlic powder per 1000 g diet; G20: 20 g garlic powder per 1000 g diet; G30: 30 g garlic powder per 1000 g diet.

**Table 6:** Serum biochemistry of Eurasian perch provided feed supplemented with garlic powder.

							Linear Trend		Quadratic Trend	
Parameters	Control	G10	G20	G30	ANOVA	*ƞ*_p_^2^	*P*-Value	R^2^	*P*-Value	R^2^
Alanine aminotransferase (ukat L^−1^)	0.28 ± 0.10	0.27 ± 0.07	0.28 ± 0.08	0.19 ± 0.09	0.27					
Aspartate aminotransferase (ukat L^−1^)	1.39 ± 0.89	1.92 ± 1.26	1.83 ± 1.42	0.75 ± 0.40	0.24					
Cholesterol (mmol L^−1^)	8.28 ± 1.43 ^b^	6.02 ± 1.13 ^a^	7.01 ± 0.61 ^a^	6.27 ± 0.44 ^a^	0.00					
Triglycerides (mmol L^−1^)	9.28 ± 1.94	9.79 ± 6.20	14.59 ± 4.41	11.30 ± 3.63	0.17					
Albumin (g L^−1^)	11.16 ± 0.77 ^b^	13.10 ± 1.28 ^c^	12.83 ± 1.47 ^c^	9.53 ± 1.28 ^a^	0.00	0.61	0.00	0.55	0.00	0.55
Total protein (g L^−1^)	43.18 ± 3.52	42.86 ± 2.26	43.46 ± 1.94	41.51 ± 1.35	0.52					

Values are presented as (mean ± SD; *n* = 6). Mean values with different superscripts within a row vary significantly according to one-way ANOVA (*p* < 0.05). R^2^ = R square. *ƞ*_p_^2^ = partial eta squared. Control: without garlic supplement; G10: 10 g garlic powder per 1000 g diet; G20: 20 g garlic powder per 1000 g diet; G30: 30 g garlic powder per 1000 g diet.

**Table 7:** Immunological parameters of Eurasian perch provided feed supplemented with garlic powder.

							Linear Trend		Quadratic Trend	
Parameters	Control	G10	G20	G30	ANOVA	*ƞ*_p_^2^	*P*-Value	R^2^	*P*-Value	R^2^
Respiratory burst activity (MFI)	9290.33 ± 1185.85	7873.66 ± 1267.97	7688.00 ± 1675.28	10,838.00 ± 4639.88	0.60					
Lymphocytes phagocytic activity%	42.10 ± 8.51	46.81 ± 7.86	40.25 ± 10.62	41.87 ± 6.24	0.57					
Lymphocytes phagocytic index	14.45 ± 2.70	14.43 ± 4.86	14.14 ± 5.16	14.47 ± 5.25	0.99					
Myeloid phagocytic activity%	52.95 ± 7.69	48.03 ± 8.25	54.08 ± 10.61	52.20 ± 6.78	0.63					
Myeloid phagocytic index	16.15 ± 2.50	19.41 ± 7.48	17.37 ± 7.43	17.91 ± 6.97	0.85					

Values are presented as (mean ± SD; *n* = 6). R^2^ = R square. *ƞ*_p_^2^ = partial eta squared. Control: without garlic supplement; G10: 10 g garlic powder per 1000 g diet; G20: 20 g garlic powder per 1000 g diet; G30: 30 g garlic powder per 1000 g diet. MFI: Mean fluorescence intensity.

## Data Availability

The data presented in this research is available on request from the corresponding author.
